# Two-Pronged Treatment of Hemoperitoneum and Abnormal Uterine Bleeding in an Adolescent Girl With Congenital Fibrinogen Deficiency

**DOI:** 10.3389/fmed.2020.00181

**Published:** 2020-05-21

**Authors:** Yiran Wang, Lanbo Zhao, Xue Feng, Qing Li, Sijia Ma, Dongxin Liang, Mingge Liu, Panyue Yin, Qiling Li, Qinrui Lu

**Affiliations:** ^1^Department of Obstetrics and Gynecology, The First Affiliated Hospital of Xi'an Jiaotong University, Xi'an, China; ^2^Guipei 77, Health Science Center, Xi'an Jiaotong University, Xi'an, China

**Keywords:** CFD, hypermenorrhea, hemoperitoneum, AUB, LNG-IUS, COC

## Abstract

**Introduction:** If a woman suffers from congenital fibrinogen deficiency (CFD), she might undergo hypermenorrhea monthly and possibly to suffer from hemoperitoneum due to a ruptured follicle or corpus luteal cyst every month, which seriously threaten her health and quality of life. Here, we creatively used a combination of the levonorgestrel-releasing intrauterine system (LNG-IUS) and the combined oral contraceptives (COC) for a girl with CFD.

**Case presentation:** A 14-year-old girl presented with no obvious cause of persistent and severe lower abdominal pain that began 8 h prior. After examination, she was diagnosed as hemoperitoneum. CFD was diagnosed when she was 2 years old and she had two hospitalizations due to hemorrhagic anemia caused by menorrhagia. Therefore, after successful conservative treatment of hemoperitoneum, a combination of LNG-IUS and COC was used for the long-term conservative management of hypermenorrhea and hemoperitoneum. During the half-year of follow-up, she had hypomenorrhea without hemoperitoneum.

**Conclusions:** To the best of our knowledge, this was the first patient treated with such a procedure in the literature, and we recommend every woman with CFD at puberty or reproductive age receives this two-pronged treatment.

## Introduction

Fibrinogen and fibrin play important and overlapping roles in blood clotting and fibrinolysis (1). Congenital fibrinogen deficiency (CFD) is a rare inherited autosomal recessive disease with a prevalence of 1 in 1,000,000 (2). Only a few known cases are reported in the literature. CFD is caused by various mutations in three fibrinogen genes, affecting both the quantity and quality of circulating fibrinogen, the blood coagulation factor (1). Hemorrhage easily occurs in CFD patients; bleeding often happens in the skin, mucous membrane, genitourinary system, digestive system, and other regions (3).

In CFD patients, hypermenorrhea is the most common gynecological complication. The incidence exceeds 70% (4), seriously affecting patient's quality of life and eventually leading to severe anemia, damaging multiple organs throughout the body. Regarding hemoperitoneum due to ovulation, since it was first reported in 1981, only seven cases have been reported in the literature (5–11). Hemoperitoneum is rare but cannot be ignored. Once it occurs, it can be serious and life-threatening requiring surgical interventions and even oophorectomy. In the seven reported cases, whether or not they underwent surgery, some CFD patients did not receive secondary treatment, resulting in rehospitalization for hemoperitoneum. Some patients used combined oral contraceptives (COC) to inhibit ovulation, but when they stopped taking their drugs or forgot to take them, hemoperitoneum occurred again. The success rate for treating menorrhagia with COC is only 36.8% (12) to 68% (13). Therefore, we combined levonorgestrel-releasing intrauterine system (LNG-IUS) and COC for our patient to reduce her menstrual amount more effectively and prevent hemoperitoneum simultaneously.

## Case Presentation

A 14-year-old girl presented to the Department of Gynecology of the First Affiliated Hospital of Xi'an Jiaotong University with no obvious cause of persistent and severe lower abdominal pain that began 8 h prior. After receiving 450 mL of plasma at another hospital, she was referred to our facility since her test results suggested that her fibrinogen was only 0.10 g/L. She developed spontaneous epistaxis bleeding 7 days after she was born. At 2 years of age, CFD was diagnosed. She had a history of spontaneous auditory canal bleeding at 7 years of age. Her menarche took place at the age of 13, every menstrual period she needed to use more than 40 sanitary pads, accompanied by fatigue, rapid heartbeat, and unable to attend physical education classes or daily physical exercise, showing that she may have hypermenorrhea. She even had two hospitalizations due to hemorrhagic anemia caused by menorrhagia 9 and 3 months prior. But no medical treatment was performed afterwards. Upon presentation at our hospital, she was in the middle of her menstrual cycle. Her abdominal pain was accompanied by nausea, vomiting, dizziness, and palpitation. Besides, she denied the history of smoking.

After admission to the Department of Gynecology, we had a detailed physical examination. She was 155 cm and weighed 50 kg. Her vital signs exhibited blood pressure of 107/74 mmHg, a heart rate of 105 beats/min, a respiration rate of 23 breaths/min, and the body temperature of 36.5°C. General examination revealed marked pallor, but the mind was clear and no bleeding spots on skin. Abdominal examination revealed a soft abdomen without tenderness or rebound pain, but the mobile voiceless was positive. No obvious abnormalities were found in other physical examinations. Since the girl and her parents firmly asserted a history of asexual life, we didn't perform a gynecological examination.

Then the patient underwent transabdominal ultrasound demonstrating that a visible 3.1 cm × 2.4 cm cystic mass next to her right ovary. The mass boundary was clear and the morphology was regular. No obvious blood flow signal was observed. The fluid dark area in front of her uterus was approximately 8.3 cm × 8.0 cm, and the fluid dark area of her uterus rectal lacuna was 5.5 cm × 2.8 cm, where sparse spot reflection was visible. The right iliac fossa level was 3.9 cm, and that of the left iliac fossa was 2.0 cm. There was no abnormality in the appendix.

The patient's coagulation function examination revealed signs of difficult coagulation. Her prothrombin time was 23.30 s (normal: 11–14 s), prothrombin activity was 37% (normal: 84–128%), international standard ratio was 2.07 (normal: 0.94–1.3), activated partial thromboplastin time was 43.8 s (normal: 28–43.5 s), thrombin time was 33.3 s (normal: 14.0–21.0 s), fibrinogen was 0.30 g/L (normal: 2–4 g/L). Her hemoglobin was 71 g/L.

When the girl was admitted to our hospital due to abdominal pain, we considered it may be ruptured corpus luteum, ruptured follicles, ectopic pregnancy, torsion of the ovarian cyst or appendicitis. Since the girl was too young, and deny the history of sex life resolutely, we ruled out the possibility of ectopic pregnancy. Her abdominal pain was not metastatic, and there were no signs of peritoneal irritation. Ultrasound showed the appendix was normal, so appendicitis can also be ruled out. Ultrasound images confirmed the presence of hemoperitoneum, so the possibility of torsion of ovarian cyst pedicles was also low, thus the initial judgment was ruptured corpus luteum or ruptured follicles. The complete diagnosis of the girl included: hemoperitoneum (ruptured corpus luteum? ruptured follicles?), CFD, abnormal uterine bleeding-coagulation (AUB-C), and hemorrhagic anemia (moderate).

**Figure 1:**
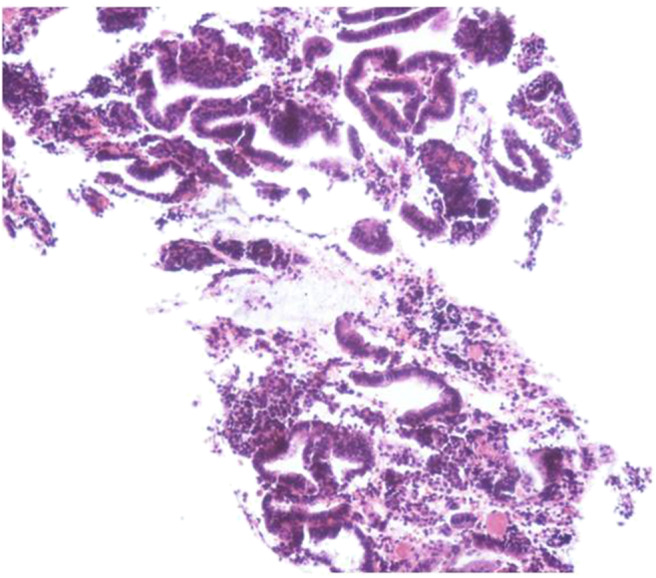
After hematoxylin and eosin (HE) immunostaining, microsection showed the endometrium comprised of proliferative endometrial cells.

Considering the patient's vital signs were stable, based to the advice of a Department of Hematology consultation, we utilized conservative treatment including rehydration support, aminocaproic acid hemostatic treatment, 2 units of red blood cell suspension, 200 mL of frozen plasma, and 6 g of fibrinogen. After 3 days of hospitalization, her fibrinogen reached 0.50 g/L. We then placed a LNG-IUS (52 mg/piece) under intravenous anesthesia. Diagnostic curettage was performed simultaneously. The pathological results suggested that it was the proliferative phase of the uterus (Figure 1), it also clarified that this hemoperitoneum was caused by ruptured follicles rather than the corpus luteum. The patient received COC (30 μg ethinylestradiol with desogestrel, 30 DSG) on the day of surgery, and then one tablet at approximately the same time every day for 63 consecutive days. When her anemia was corrected, COC administration was changed to 21 days of continuous medication as usual.

## Discussion

In the seven CFD cases reported with hemoperitoneum (Table 1) (5–11), the age of first onset ranged from 14 to 24 years, with an average age of 18.71 years. Six cases (85.71%) had a history of bleeding. Two cases (28.57%) underwent simple ovarian rupture repair, three cases (42.86%) underwent unilateral oophorectomy, and one case (14.29%) underwent bilateral oophorectomy. No surgery was performed in one case (14.29%). COC was used in five cases (71.43%), of which three cases (60%) of hemoperitoneum occurred again after withdrawal or missed administration. The number of hospitalizations dues to hemoperitoneum more than or equal to twice was four cases (57.14%).

**Table 1:** Review of the literature on hemoperitoneum in patients with congenital fibrinogen deficiency.

**References**	**Age (years)**	**Previous bleeding episodes**	**HB (g/dL)**	**FIB (mg/dl)**	**Received surgery**	**Follow-up treatment**	**Prognosis**
Bottiniet al. (6)	15 (1st Episode)	Menorrhagia complicated with anemia	–	–	Removed the peritoneal blood	No	5 months later, hemoperitoneum occurred again
	15 (2nd Episode)		–	–	Right oophorectomy	COC	1 years later, hemoperitoneum occurred again for dismissed COC
	16 (3rd Episode)		–	–	Left oophorectomy	-	–
Castaman et al. (7)	14 (1st Episode)	Umbilical stump bleeding and spontaneous hemartrosis	–	–	Right oophorectomy	No	4 years later hemoperitoneum occurred again
	18 (2nd Episode)		–	–	Simple ovarian rupture repair	COC	Without further episodes of hemoperitoneum over a 5-year follow-up period
Cetinkaya et al. (9)	24 (1st Episode)	No	8.8	0	Simple ovarian rupture repair	COC	3 months later hemoperitoneum occurred again, due to stopped COC since she wished to conceive.
	24 (2nd Episode)		9.8	0	No	–	–
Kim et al. (11)	20	Epistaxis	10.3	15	Simple ovarian rupture repair	COC	–
Koussi et al. (8)	14	Cephalhematoma	12.3	0	No	No	–
Ozdemir et al. (10)	22 (1st episode)	–	7.5	–	No	No	1 years later hemoperitoneum occurred again
	23 (2nd episode)		4.8	–	Right ovary cystectomy	No	3 months later, hemoperitoneum occurred again
	23 (3rd Episode)		9.8	–	No	No	1 years later hemoperitoneum occurred again
	24 (4th episode)		5.5	–	Right Salpingo-oophorectomy	COC	4years later hemoperitoneum occurred again due to stopped COC since she wished to conceive.
	28 (5th episode)		9.9	–	No	–	–
Schneider et al. (5)	22	Spontaneous rupture of a histologically normal spleen	10.3	0	Left oophorectomy	–	–

*HB, Hemoglobin; FIB, Fibrinogen; COC, Combined oral contraceptives; –, Not reported*.

Since hemoperitoneum caused by ovulation may recur every month, most doctors (6–11) prevent it by putting their patients on long-term COC therapy for its double benefits. On the one hand, the estrogen component in COC prevents follicle-stimulating hormone secretion and the development of a dominant follicle, and the progestogen prevents the luteinizing hormone surge and ovulation, which will avoid the rupture of the follicle and corpus luteum from the root cause. On the other hand, the progestogen creates an atrophic endometrial lining, that reduces overall blood loss at the time of menstruation (14). Regarding ovulation suppression, COC can achieve significant results. However, the effect of reducing menstruation is unsatisfactory: the treatment success rate is from 36.8% (13) to 68% (14). In our case, the patient's repeated history of hypermenorrhea with hospitalization discouraged her parents from assuming such a high risk of failure, whether it was for economic or spiritual considerations, they refused to try COC treatment and planned to have her uterus removed to completely cure her AUB-C. Although almost 90% of pregnancies are reportedly complicated by recurrent first trimester abortion, placental abruption, and postpartum hemorrhage (15), after strict pregnancy management, cases of successful pregnancy have also been reported (16–19). However, hysterectomy would forever deprive our patient of childbearing. According to the relevant literature (13, 14, 20, 21), LNG-IUS is more effective than COC at reducing overall menstrual blood loss and reaching menstrual “normality.” Therefore, after full communication with her family about the related advantages and complications, LNG-IUS combined with COC was used for the patient's long-term management. Though the girl wasn't obese, had no history of smoking, and fibrinogen in plasma was rare, the risk of thrombosis was lower than that of the average person, to be more cautious, we chose 30DSG which was low thrombosis risk (22) for her. This treatment not only alleviates her hypermenorrhea and prevented hemoperitoneum, but also it has an additional advantage that when she wishes to conceive, she will have to come to the hospital for LNG-IUS removal. Thus, she can be closely monitored during pregnancy preparation rather than stop taking COC herself.

A network follow-up was conducted when the first withdrawal bleeding occurred 2 months after the patient was discharged from the hospital. She had no abdominal pain and the number of sanitary pads used had dropped to 23 pieces, using the pictorial blood loss assessment chart (PBAC) (23) to assess her menstrual flow, the PBAC scoring < 100 that meant normal menstrual flow. Two months later, after she finished the second cycle of medication, she returned to the clinic for reexamination. Her face was obviously ruddy and her hemoglobin had increased to 120 g/L. Thus, the COC was returned to the regular 21 days of continuous medication. Her sanitary pads usages were approximate 20 pieces, and PBAC scores were less than 100 in the next two menstrual cycles. Although we couldn't get accurate PBAC scores before her treatment, it can be proved from the decrease in the number of sanitary pads used that our treatment was very feasible.

Both COC and LNG-IUS are common methods for ordinary being to treat AUB, COC monotherapy was used for the long-term management of CFD patients, in the currently reported literature. Since CFD patients usually experience hypermenorrhea shortly after menarche, at that time, almost all of them are minors who have no sex life; doctors will be more conservative than reproductive women when choosing treatments. However, the high failure rate of COC to treat of hypermenorrhea continues to put patients at risk. In our case, after full communication with the girl and her parents, we implant a LNG-IUS for the girl besides taking COC. A long follow up still necessary to evaluate the impact and tolerance of the LNG-IUS, but for the moment, our case had proved that COC combined with LNG-IUS can be chose when needed.

## Data Availability Statement

The raw data supporting the conclusions of this article will be made available by the authors, without undue reservation, to any qualified researcher.

## Ethics Statement

Written informed consent was obtained from the girl's parents for the publication of any potentially identifiable images or data included in this article.

## Author Contributions

QilL conceptualized the case report. YW, LZ, XF, and QinL collected the history. YW wrote the first draft of the manuscript. SM, DL, ML, PY, and QirL revised the manuscript. All authors contributed to the revision of the manuscript and approved its final version.

## Conflict of Interest

The authors declare that the research was conducted in the absence of any commercial or financial relationships that could be construed as a potential conflict of interest.
